# Isolation and phylogenetic analysis of orf virus from the sheep herd outbreak in northeast China

**DOI:** 10.1186/1746-6148-8-229

**Published:** 2012-11-23

**Authors:** Wei Li, Zhangyong Ning, Wenbo Hao, Deguang Song, Feng Gao, Kui Zhao, Xiaoqing Liao, Ming Li, Daniel L Rock, Shuhong Luo

**Affiliations:** 1 Institute of Antibody Engineering, School of Biotechnology, Southern Medical University, N. Guangzhou Avenue, 1838, Guangzhou, 510515, People’s Republic of China; 2 College of Veterinary Medicine, South China Agricultural University, Guangzhou, 510642, People’s Republic of China; 3 College of Animal Science and Veterinary Medicine, Jilin University, Changchun, 130062, China; 4 Department of Pathobiology, College of Veterinary Medicine, University of Illinois at Champaign-Urbana, S. Lincoln Avenue, 2001, Urbana, IL 61802, USA

**Keywords:** Parapoxvirus, Orf virus, Phylogenetic analysis, Sheep, Zoonosis, nMDS

## Abstract

**Background:**

Orf is a zoonotic and epitheliotrophic contagious disease that mainly affects sheep, goats, wild ruminants, and humans with a worldwide distribution. To date, there is little information on the characterization of ORFV strains that are endemic in Mainland China. In addition, the relationship between the severity of disease and the molecular profile of ORFV strains has not been fully elucidated.

**Results:**

From the recent outbreak of a sheep herd in Nongan, northeast of China, the novel orf virus (ORFV) strain NA1/11 was successfully isolated. Western blot analysis indicated that the NA1/11 strain cross reacts with monoclonal antibody A3 and infected sheep ORFV antiserum. The purified virions revealed the typical ovoid shape when observed by atomic force microscopy. To determine the genetic characteristics of the NA1/11 strain, the sequences of ORFV011 (B2L), ORFV059 (F1L), ORFV109, ORFV110 and ORFv132 (VEGF) genes were amplified and compared with reference parapoxvirus strains. Non-metric multidimensional scaling (nMDS) was performed to analyze the nucleotide similarities between different ORFV strains.

**Conclusions:**

Phylogenetic analysis based on ORFV 011 nucleotide sequences showed that the NA1/11strain was closely related to Xinjiang and Gansu strains. ORFV110 and ORFV132 genes are highly variable. The results revealed that precise phylogenetic analysis might provide evidence for genetic variation and movement of circulating ORFV strains in Northeast China. In addition, nMDS analysis showed that geographic isolation and animal host are likely major factors resulting in genetic differences between ORFV strains.

## Background

Orf, also known as contagious pustular dermatitis or contagious ecthyma, is an epitheliotrophic disease that mainly affects sheep, goats, wild ruminants, and humans with a worldwide distribution [1-3]. The lesions are characterized by maculopapular, vesicular pustules that mainly affect the skin around the lips, mouth muzzle, nostrils, teats, and oral mucosa and rarely extend into the esophagus, stomach, intestine, or the respiratory tract of sheep, goats and wild animals [3-6]. In humans, the most common lesions are self-limiting, painful pustules on the hands and fingers [3,4]. The infective lesions are usually confined to the areas surrounding the virus entry sites. The development stages include erythema, vesicles, pustules, and scabs [6,7]. Primary lesions are usually resolved within 1–2 months, however repeated and persistent infections can occur [6,8,9]. The mortality rate related to this disease is usually low but it can reach 93% in lambs with secondary bacterial or fungal infections [4,5]. To diagnose this disease, virus isolation is thought to be the gold standard, although gross clinical signs can be used as a good reference [10].

ORFV, the causative agent of orf, is the prototype member of the genus Parapoxvirus belong to the subfamily Chordopoxvirinae of the Poxviridae. This genus also includes pseudocowpox virus (PCPV) and bovine papular stomatitis virus (BPSV) in cattle, squirrel parapoxvirus (SPPV), and parapoxvirus of red deer in New Zealand (PVNZ) [11,12]. Parapoxviruses are ovoid in shape, with a crisscross patterned tubule-like structure on the particle surface [13]. The ORFV genome consists of linear double-stranded DNA about 138kb long with ~ 64% G+C content, which contains 132 putative genes that included 89 highly conserved genes and some variable genes [13,14]. Currently, there are four ORFV strains that have been isolated and completely sequenced, OVIA82 and OV-SA00 [14] in America, NZ2 in New Zealand [13], and D1701 in Germany [15,16].

The entire genomic sequence of the orf virus from mainland China is not available. Only partial sequences are listed in the GenBank database and phylogenetic analysis was based on the highly conserved genes of ORFV011 (B2L) and/or ORFV059 (F1L) [5,12,17]. Similarly, almost all the phylogenetic analysis data published worldwide are based on the highly conserved genes except for where the whole genomic sequence is available (Table 1). There is little information on the characterization of ORFV strains that are endemic to mainland China. In addition, the relationship between disease severity and the molecular profile of ORFV strains has not been fully elucidated.

**Table 1:** Orf virus NA1/11 and published Parapoxvirus strains used for phylogenetic analysis

**Geninfo Identifier (gi)**	**Strain**	**Species of origin**	**Country of origin**	**Genes**	**Reference**	**No. In nMDS**
294653624	JS04	Sheep	China	ORFV011,ORFV059	Liu et al. (2007)[17]	1
336169664	Gansu/2009	Sheep	China	ORFV011	Zhang et al. (unpublished)	2
269854030	Jilin	Sheep	China	ORFV011, ORFV059	Zhao et al. (2010)[5]	3
323364296	Shanxi	Goat	China	ORFV011, ORFV059	Shi (unpublished)	4
284178603	Hub/2009	Goat	China	ORFV011	Zhang et al. (2010)[12]	5
336169666	LiaoNing/10	Goat	China	ORFV011	zhang et al. (unpublished)	6
365266883	Xinjiang	Sheep	China	ORFV011	Lui et al. (unpublished)	7
365266887	Shanxi/11	Goat	China	ORFV011	Lui et al. (unpublished)	8
NA*	**NA1/11**	**Sheep**	**China**	**ORFV011, 059, 109, 110**	**Present study**	**9**
197344484	Hoping	Goat	Taiwan	ORFV011	Chan et al. (2009)[18]	10
114225403	Natou	Goat	Taiwan	ORFV011	Chan et al. (2007)[10]	11
163860191	Taiping	Goat	Taiwan	ORFV011	Chan et al. (2007)[10]	12
255040176	Korea	Goat	Korea	ORFV011	Oem et al. (2009)[19]	13
82570507	India67/04	Sheep	India	ORFV011	Hosamani et al. (2006)[11]	14
284159117	Mukteswar/09	Sheep	India	ORFV011	Veskatesan et al.(unpublished)	15
82570509	India79/04	Sheep	India	ORFV011	Hosamani et al. (2006)[11]	16
82570503	India82/04	Goat	India	ORFV011	Hosamani et al. (2006)[11]	17
82570505	India59/05	Goat	India	ORFV011	Hosamani et al. (2006)[11]	18
371925323	Assam/10	Capra	India	ORFV011	Bora et al. (unpublished)	19
357595057	Assam/09	Capra	India	ORFV011	Bora et al. (unpublished)	20
308225028	Muk/2000	Goat	India	ORFV011	Bora et al. (unpublished)	21
367462731	MT-05	Sheep	Brazil	ORFV011	Abrahao et al. (unpublished)	22
344050163	NE2	Goat	Brazil	ORFV011	Abrahao et al. (unpublished)	23
367462733	NE1	Goat	Brazil	ORFV011	Abrahao et al. (unpublished)	24
344050167	A	Goat	Brazil	ORFV011	Abrahao et al. (unpublished)	25
344050165	D	Sheep	Brazil	ORFV011	Abrahao et al. (unpublished)	26
325073632	D1701	Goat	Germany	ORFV011, 059, 109, 110	Mayr (1981)[15]; McGuire(2012)[16]	27
74230714	NZ2	Sheep	New Zealand	ORFV011, 059, 109, 110	Mercer et al. (2006)[13]	28
40019123	OV-SA00	Sheep	USA	ORFV011, 059, 109, 110	Delhon et al. (2004)[14]	29
40019122	OV-IA82	Sheep	USA	ORFV011, 059, 109, 110	Delhon et al. (2004)[14]	30
37594893	NA	Musk ox	USA	ORFV011	Guo et al. (2004)[20]	31
37594897	NA	Takin	USA	ORFV011	Guo et al. (2004)[20]	32
37594895	NA	Sheep	USA	ORFV011	Guo et al. (2004)[20]	33
33415066	NA	Goat	USA	ORFV011	Guo et al. (2003)[20]	34
33415068	vaccine	Goat	USA	ORFV011	Guo et al. (2003)[8]	35
345843287	F07.821R	Reindeer	Finland	ORFV011	Hautaniemi et al. (2011)[21]	36
345843285	F07.816R	Reindeer	Finland	ORFV011	Hautaniemi et al. (2011)[21]	37
345843283	F07.810R	Reindeer	Finland	ORFV011	Hautaniemi et al. (2011)[21]	38
345843281	F07.808R	Reindeer	Finland	ORFV011	Hautaniemi et al. (2011)[21]	39
345843277	F94.848R	Reindeer	Finland	ORFV011	Hautaniemi et al. (2011)[21]	40
345843279	F92.849R	Reindeer	Finland	ORFV011	Hautaniemi et al. (2011)[21]	41
345843289	F07.3748S	Sheep	Finland	ORFV011	Hautaniemi et al. (2011)[21]	42
345843291	F09.1160S	Sheep	Finland	ORFV011	Hautaniemi et al. (2011)[21]	43
345843271	F07.801R	Reindeer	Finland	ORFV011	Hautaniemi et al. (2011)[21]	
255761105	Jodhpur	Camel	India	ORFV011	Lucinda et al. (2010)[22]	
295646610	Cam/09	Camel	India	ORFV011	Venkatesan et al. (unpublished)	
345843273	F05.990C	Bovine	Finland	ORFV011	Hautaniemi et al. (2011)[21]	
345843275	F10.3081C	Bovine	Finland	ORFV011	Hautaniemi et al. (2011)[21]	
345843269	F07.798R	Reindeer	Finland	ORFV011	Hautaniemi et al. (2011)[21]	
37594899	PCV	NA	USA	ORFV011	Guo et al. (2004)[20]	
37594901	BPSV	NA	USA	ORFV011	Guo et al. (2004)[20]	
40019124	BV-AR02	Cattle	USA	ORFV011, 059, 09, 110	Delhon et al. (2004)[14]	

NA: Not available. Virus strains above F07.801R were applied to pairwise distance calculations and nMDS analysis.

Considering heterogeneity of different ORFV field strains, we focused on isolation and characterization of ORFV from lesions during an epidemic of multifocal, persistent, severe, proliferative dermatitis in lambs in China. The novel NA1/11 strain was successfully isolated and characterized by Western blotting, electronic morphological observation, and polymerase chain reaction analysis. Genetic diversity was also determined by comparing the full lengths of ORFV 011, 059, 109, 110 and 132 genes with reference strains in the literature. This study on the genetic diversity of ORFV in China may contribute to an improved understanding of ORFV pathogenesis, infection biology and epidemiology, and facilitate ORFV vaccine development.

Ordination is a method helpful to data clustering in multivariate analysis. Non-metric multidimensional scaling (nMDS) is an ordination technique similar to principal component analysis (PCA). Ordination can avoid constructing linear relationships. It was used in this study to visualize and analyze the relationship between genetic distances and distances in species or geographic space.

## Results

### Clinical gross pathological changes

**Figure 1:**
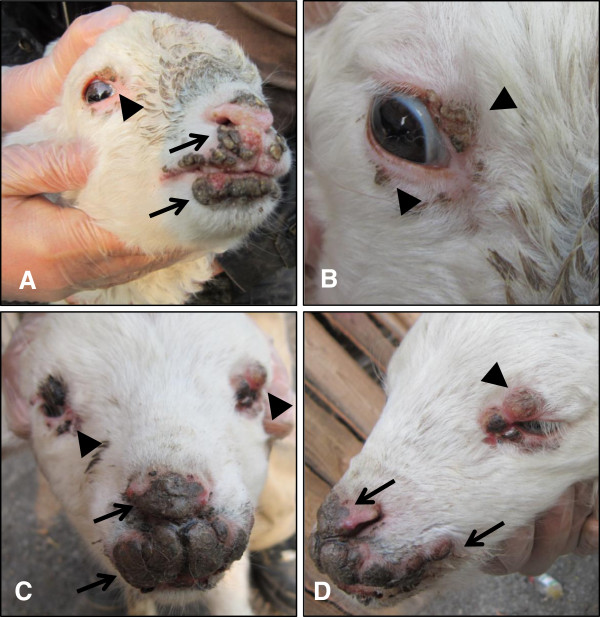
**Typical clinical signs of orf virus infection in sheep.** Proliferative lesions on the skin of the lips around the mouth, nostrils (arrows), and the eyelids (arrowheads).

In the sheep herd in Nongan clinical lesions with characteristic symptoms of orf such as papules, pustules, and scabs were recorded in 11 lambs from the flock. Two lambs showing proliferative lesions on the lips, nostrils, and eyelids were examined (Figure 1). Both lambs presented with weight loss, anorexia, and proliferative papillomatous nodules ranging from 3–8 cm in diameter. Of the 11 infected lambs, 10 recovered about 30 days after clinical signs first appeared. One two-month old lamb died of secondary infection. The morbidity and mortality of the outbreak were 9.6% (11 out of 115) and 0.9% (1 out of 115) respectively. No cases of disease were recorded in breed ewes or the farm staff.

### Orf virus isolation and purification

**Figure 2:**
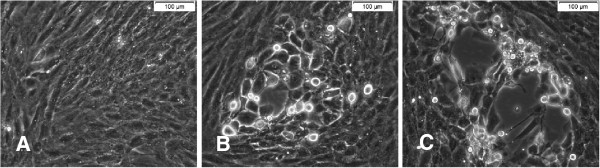
**Cytopathic effect (CPE) of the orf virus strains NA1/11 in primary ovine fetal turbinate (OFTu) cells.** Uninfected OFTu cells (**A**), 24 hpi (**B**) and 36hpi (**C**) of NA1/11 in OFTu cells. Scale bars = 100 μm.

All the homogenates from tissues collected from the outbreak were inoculated into cultures of OFTu cells. The cytopathic effect (CPE), indicated by cell rounding, pyknosis, and cell detachment, was observed in cell cultures but not in mock-infected cells. CPE was evident 1 to 2 days after the third blind passage (Figure 2). To isolate a single clone of the viral strain, a plaque assay was performed to isolate a monoclonal plaque, caused by a single virus. The isolated virus from the single plaque was amplified, titrated, and stored at −80°C. The virus isolate was designated ORFV/Nongan-C1/2011 (NA1/11). The mature virions were purified by sucrose gradient ultra-centrifugation. A major virus band was obtained after centrifugation of virus infected OFTu cells in the 32%–36% sucrose gradient.

### Western blot analysis

**Figure 3:**
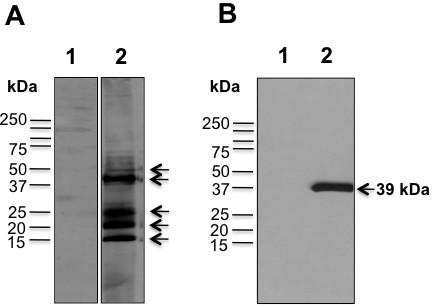
**Western blot analysis of cross reaction of the NA1/11 strain with ORFV-positive antiserum and monoclonal antibodies A3 against ORFV-Jilin ORFV059.** 2 μg of the purified viral protein or 20 μg of OFTu total cell lysate were resolved by using SDS-PAGE, transferred to nitrocellulose paper, probed with infected sheep serum (**A**) and Mab A3 against ORFV-Jilin ORFV059 (**B**). The strongest reactive protein bands were 39, 25, 20 and 15 kDa but 28, 35, 49, 55, 60 and 90 kDa bands were detectable (A, Lane 2) The location of an approximately 39 kDa protein corresponding in molecular weight to ORFV059 protein is shown (B, Lane 2). The lane 1 of A and B is protein marker.

The cross reaction of the NA1/11 strain with sheep serum and monoclonal antibodies against other ORFV strains was determined using Western blot analysis. As shown in Figure 3A, the purified viral protein interacted with ORFV-positive antiserum, which was collected from a separate orf outbreak [5]. The strongest reactive protein bands were 39, 25, 20 and 15 kDa but 28, 35, 49, 55, 60 and 90 kDa bands were also detectable (Figure 3A, Lane 2). In addition, a 39 kDa protein was detected when the NA1/11 viral protein blot was incubated with Mab A3 against ORFV-Jilin ORFV059 protein (Figure 3B, Lane 2) [23], suggesting a cross reaction between NA1/11 and ORFV-Jilin strain.

### Morphological determination of the NA1/11 strain

**Figure 4:**
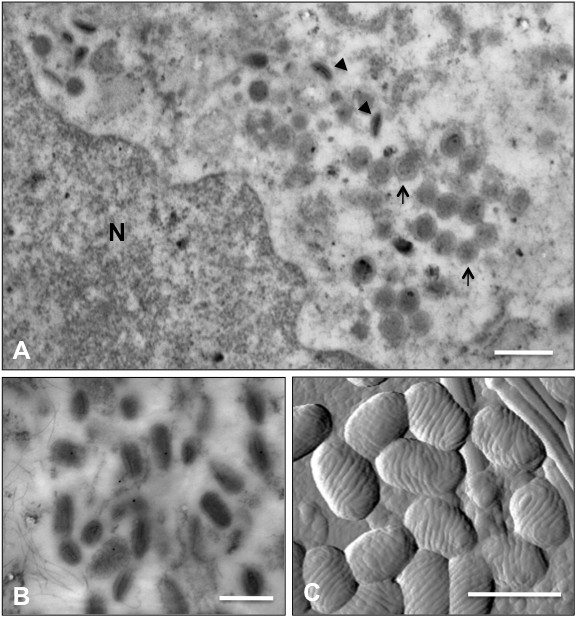
**Electron microscopy of ORFV strain NA1/11.** OFTu cells were infected with NA1/11. After 24 h, cells harvested and fixed for embedding were prepared for transmission electron microscopy as described under Materials and Methods. Fields show predominantly immature virions (**A**), intracellular mature virions (**B**) and extracellular virions under atomic force microscopy (**C**). Scale bars = 500 nm.

Two types of orf virus particles were visualized by transmission electron microscopy of ultra thin sections: the immature form (Figure 4A, arrows) and mature form (Figure 4A arrowheads and B). The NA1/11 viral particles consisted of complete, ovoid-shaped virions characteristic of parapoxvirus with a crisscross patterned tubule-like structure measuring about 260 nm×150 nm on the particle surface when observed under the MFP-3D atomic force microscope (Figure 4C). No other viruses were detected.

### PCR amplification

**Figure 5:**
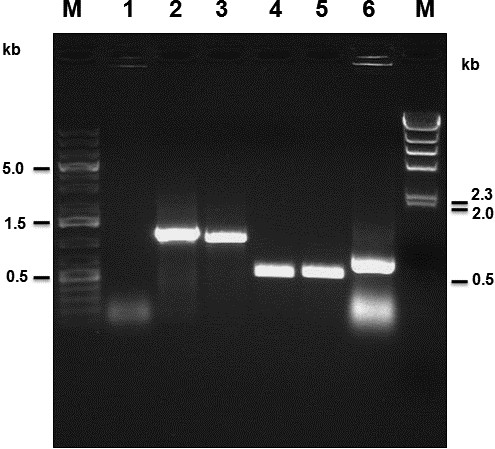
**PCR amplification of ORFV011 (B2L), ORFV059 (F1L), ORFV109, ORFV110 and ORFV132 of ORFV strain NA1/11.** The complete coding regions of ORFV011 (lane 2), ORFv059 (lane 3), ORFV109 (lane 4), ORFV110 (lane 5) and ORFV132 (lane 6) were amplified from genomic DNA isolated from NA1/11. Lane 1: DNA extracted from non-infected cells as negative control. Lane M (left): 1 kb DNA ladder (Fermentas); M (right): λ/HindIII (Invitrogen).

DNA template for PCR was prepared from tissue suspensions, infected cell culture supernatant, or purified viral particles. DNA from non-infected OFTu cells was used as a negative control. The full length of the coding regions of ORFV011, 059, 109, 110 and 132 –134 gene coding regions were amplified by PCR from genomic DNA extracted from tissue suspensions, infected cell culture supernatant, or purified viral particles but not from non-infected controls. The sizes of products were 1137, 1017, 615, 628 and 721 bp (this DNA fragment covers the full length of ORFV132 and 150 bp of ORFV134 coding region), respectively (Figure 5). The amplified products were purified and ligated into TA cloning vectors. At least three different clones of each gene were amplified and sequenced bi-directionally. The sequences were edited, aligned, and deposited in GenBank. Accession numbers are: ORFV011: JQ619903; ORFV059: JQ619904; ORFV109: JQ619905; ORFV110: JQ619906 and ORFV132: JQ663432.

### Phylogenetic analysis

To compare and determine the phylogenetic relationship of NA1/11 strain with other ORFV strains, PCPV and BPSV, the corresponding sequences obtained from GenBank were used in this study and are presented in Table 1.

**Figure 6:**
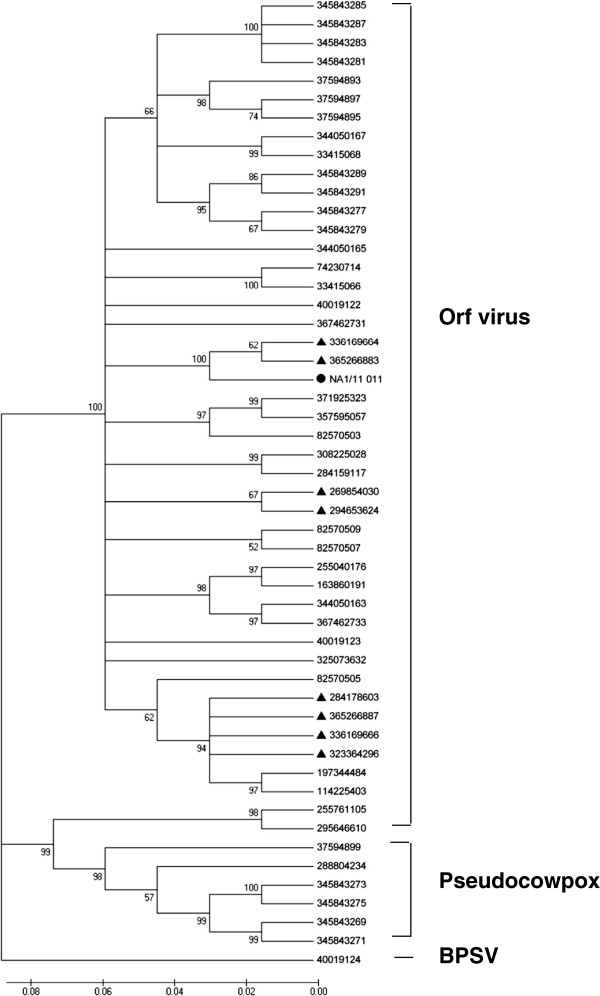
**Phylogenetic analysis based on nucleotide sequences of ORFV011.** The phylogenetic relationship was constructed by the neighbor-joining algorithm using MEGA 5.0 software; one thousand bootstrap replicates were subjected to nucleotide sequence distance (cut-off value of 50% from 1000 bootstrap replicates). All bootstrap values are displayed above branches. ·: NA1/11 isolated in this study; *black triangle*: eight different Chinese strains from database.

**Figure 7:**
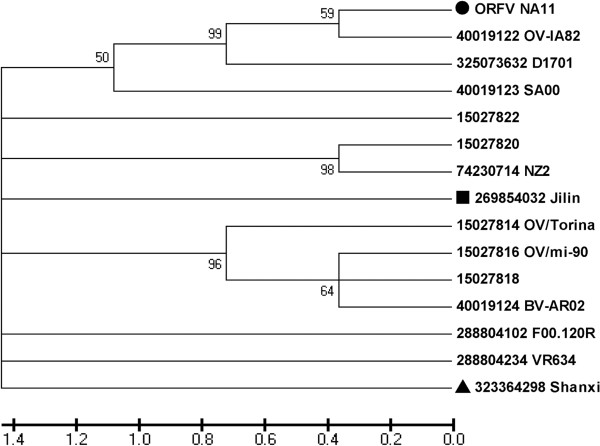
**Phylogenetic analysis based on nucleotide sequences of ORFV059.** The phylogenetic relationship was constructed by using the same method as described in Figure 6. All bootstrap values are displayed above branches. ·: NA1/11 isolated in this study.

The NA1/11-ORFV011 and 059 sequences share 96–97% and 77–99% nucleotide and 97–98% and 95–99% deduced amino acid identities with published reference ORFV strains (Table 2). The phylogenetic analysis of NA1/11-ORFV011 showed that the 9 Chinese ORFV strains cluster into three branches. NA1/11-ORFV011 clusters together with Xinjiang and Gansu strains with 99% nucleotide identities. All three Chinese strains share greater homology with Brazilian strain MT-05, American strain OV-IA82 and New Zealand strain NZ2. The ORFV011 of Jilin and Jiangsu strains cluster together with India/67/04 and Korean strains with 99% nucleotide identities. NA1/11-ORFV011 shares 97% nucleotide identities with Jilin and Jiangsu strains. The ORFV011 of Hubei, Shanxi and Liaoning strains cluster together with D1701 from Germany and OV-SA00 of TX/USA strains with 99% identity (Figure 6). NA1/11-ORFV011 shares 96% nucleotide identities with Shanxi, Shanxi/2011 and HuB/2009 strains. The identities between NA1/11-ORFV011 and PCPV or BPSV are 93% at the nucleotide level and 95 and 83% at the deduced amino acid level, respectively. The NA1/11-ORFV059 shares 75-99% nucleotide and 56-99% deduced amino acid identities with reference strains (Table 1 and 2). NA1/11-ORFV059 was close to OV-IA82 strain (Figure 7). ORFV109, ORFV110, ORFV132 are highly variable genes localized at the right terminus of the viral genome. NA1/11–ORFV109, 110 and 132 shares 88–97%, 57–96%, and 18–96% nucleotide identities and 44–88%, 40–92%, and 39–94% deduced amino acid identities with OV-IA2, NZ2, D1701, and OV-SA00 strains (Table 2). The NA1/11-ORFV109, 110, 132 and PCPV-VR634 or BPSV-BV-AR02 shares 71 or 57%, 79 or 92%, 9 or 32% nucleotide identities and 58 or 46%, 75 or 75%, 45 or 49% deduced amino acid identities, respectively (Table 2). NA1/11-ORFV109, 110, 132 shares 71%, 79%, and 9% nucleotide identities and 58%, 75%, and 45% deduced amino acid identities with PCPV-VR634 while NA1/11-ORFV109, 110, 132 shares 57%, 92%, and 32% nucleotide identities and 46%, 75%, and 49% deduced amino acid identities with BPSV-BV-AR02 (Table 2).

**Table 2:** The identity of nucleotide and amino acid sequences between the NA1/11 strain and the reference parapoxvirus strains

	**ORFV011**		**ORFV059**		**ORFV109**		**ORFV110**		**ORFV132**	
**Strain and its Geninfo Identifier (gi)**	**Nucleotide**	**Amino acid**	**Nucleotide**	**Amino acid**	**Nucleotide**	**Amino acid**	**Nucleotide**	**Amino acid**	**Nucleotide**	**Amino acid**
NZ2(74230714)	97	98	99	99	97	81	96	92	96	94
OV-IA82(40019122)	97	98	97	99	95	88	85	79	85	81
OV-SA00(40019123)	96	97	97	96	88	44	58	41	18	39
D1701(325073632)	96	96	98	95	94	48	57	40	88	80
BV-AR02(40019124)	93	83	75	56	57	46	92	75	32	49
VR634(288804234)	93	95	91	84	71	58	79	75	9	45

**Figure 8:**
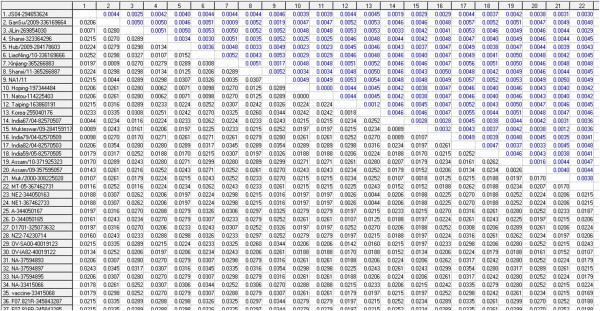
**Part of pairwise distance matrix based on nucleotide sequences of ORFV011 of 43 ORFV strains.** Pairwise distances calculations were accomplished using MEGA 5.0 software. Distances and standard errors are respectively displayed in the lower-left matrix and the upper-right matrix.

In pairwise distance matrix the three Chinese strains cluster together, the distances between NA1/11 and GanSu/2009 is 0.0044, the distance between NA1/11 and Xinjiang is 0.0035, and the distance between GanSu/2009 and Xinjiang is 0.0009 (Figure 8).

### NMDS

**Figure 9:**
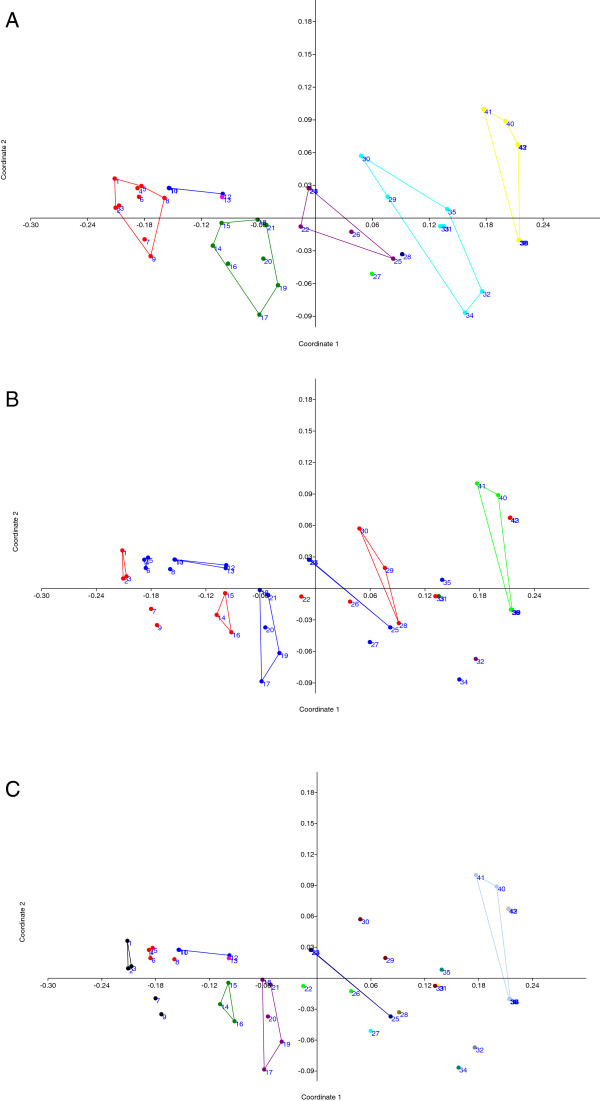
**NMDS scatter plots based on pairwise distance matrix.** Two coordinates are respectively pairwise distances and standard errors. Different colors of plots represent: **(A)** ORFV strains in different geographic isolation. (Red circle): mainland China (blue circle): Taiwan; (Fuchsia circle): Korea; (green circle): India; (purple circle): Brazil; (Lime circle): Germany; (midnight blue circle): New Zealand; (aqua circle): USA; (yellow circle): Finland. **(B)** ORFV strains with different animal host species (red circle): sheep; (blue circle): goat (including capra); (green circle): musk ox; (purple circle): takin; (lime circle): reindeer. **(C)** ORFV strains with different host species and in different geographic isolation. (black circle): sheep, mainland China; (red circle): goat, mainland China; (blue circle): goat, Taiwan; (fuchsia circle): goat, Korea; (green circle): sheep, India; (purple circle): goat (including capra), India; (lime circle): sheep, Brazil; (midnight blue circle): goat, Brazil; (aqua circle): goat, Germany; (olive circle): sheep, New Zealand; (maroon circle): sheep, USA; (yellow circle): musk ox, USA; (slate gray circle): takin, USA; (cyan circle): goat, USA; (dodger blue circle): reindeer, Finland; (gray circle): sheep, Finland.

The results of nMDS analyses are displayed as scatter plots (Figure 9). Analysis of similarities between different strains grouped by geographic isolation indicated that different strains from the same region are in close proximity, e.g. strains from China, Taiwan, India, USA, Brazil, or Finland. Strains from the same continent are closer to each other than strains from other continents except D1701 and NZ2 (Figure 9A). The similarities among different strains grouped by host species showed that strains with the same host species did not scatter or group together because of their different geographic locations. However, they did gather into clusters (Figure 9B). Interestingly, the similarities between different strains grouped by host species plus geographic isolation (Figure 9C) are similar to the pattern of in Figure 9B. Strains in the same region isolated from different hosts have a big genetic variation. In nMDS analysis NA1/11 strain clusters with the other eight strains from China (Figure 9A) and is very close in distance to strain No.7 (Xinjiang, sheep), which is in accordance with the results of phylogenetic analysis.

## Discussion

In recent years there have been increased reports of ORFV infections in sheep, goats, wild animals, and humans worldwide [5,10,12,19,20,24-27]. The clinical features of the infected animals varied from subclinical to multiple lesions around the lips, mouth, muzzle, nostrils, teats, and oral mucosa and occasionally within the buccal cavity esophagus, stomach, intestine, or the respiratory tract [3-5,24,27]. Recently genital lesions [24] and multifocal cutaneous infections [25] were also reported. However, virus isolation and characterization is seldom achieved in China. In this outbreak, the lesions were located around the mouth, muzzle, and the upper and lower eyelids (Figure 1). These are classical clinical signs and strong evidence of an orf virus infection.

The identity of NA1/11 strain was confirmed by Western blot, electron microscopy, amplification of specific genes from viral DNA by PCR, and DNA sequencing. Western blot analysis revealed four strong reactive protein bands and six weak bands detected by sheep ORFV antiserum (Figure 3). Furthermore, a 39 kDa protein was detected in the NA1/11 viral protein blot using a Mab A3 against ORFV-Jilin ORFV059 protein [23], suggesting a cross reaction between the two different isolates and providing a tool for ORFV epidemiological surveillance and diagnosis.

The observed shape of NA1/11 virus was similar to the typical ovoid-shape of orf virus and the specific PCR products from the genomic DNA of the isolated virus confirmed our diagnosis of orf infection from this sheep herd. In China, from 2005 to 2006, ORFV outbreaks occurred in the provinces of Inner-Mongolia, Guangxi, Shanxi, Fujian, Jilin, Jiangsu and Beijing, and the government strengthened the vaccination program to control this disease [12]. However, in 2010, Jilin and Hubei provinces reported outbreaks of ORFV infected sheep herds [5,12]. In 2011, we found an outbreak in sheep herds in Nongan, northeast China. Because of the persistence or repetition of ORFV infections, it is very difficult to eliminate this virus. ORFV infection is considered a re-emerging disease in China and other countries [24,25].

In the ORFV genome, ORFV011, 059, 109, 110 and 132 genes have distinct functions for orf virus infections and pathogenesis. The ORFV011 gene encodes the major envelope immunogenic protein [28], while the ORFV059 gene encodes an immunodominant protein and plays a role in virus maturation and adsorption [23]. Both of these proteins are conserved and are used for orf detection, molecular characterization, and phylogenetic analysis [5,28]. ORFV109 and 110 encode envelope type II glycoproteins, which are expressed in inter- and extracellular enveloped virions [13]. The ORFV132 protein is an apparent homolog of the mammalian vascular endothelial growth factor (VEGF) family and plays an important role in the development of lesions induced by the orf virus [29]. These three genes are highly variable. According to the phylogenetic relationships based on the ORFV011, NA1/11 clustered with two Chinese strains, Xinjiang and Gansu, isolated from Northwest China, and was close to the Brazilian strain MT-05, American strain OV-IA82 and New Zealand strain NZ2. Usually phylogenetic analysis indicates a hypothetical origin of virus strains; the results presented here suggest that the OV-IA82 and NZ2 strains were introduced into China, but it is difficult to determine the route through which the new strains entered into China. Theoretically, the diversity of viral sequences is linked to viral strain differences, or other factors such as flock immunity, genetic susceptibility, or method of exposure. This implies that we may use the NZ2 strain, which is licensed and widely used in Europe [19] to vaccinate sheep herds and reduce ORFV outbreaks in China. We propose that the phylogenetic analysis of parapoxvirus should use entire genomic sequences to obtain more precise results.

Our results indicated that the nucleotide sequences similarities (or distances) among different ORFV strains are divergent (Figure 8 and 9). We hypothesize that the genetic differences among ORFV virus strains mainly relate to geographic location and animal host. However, as the variables are non-metric, the nMDS results need further complete genome comparisons to understand fully the genetic diversity and epidemiology of this complex group of viruses.

## Conclusions

In summary, Orf infection is endemic in China. Although vaccination has been implemented in some regions to control the disease, an increase of orf infection in the dairy sheep and goat population in China has been observed in recent years, causing significant veterinary and economic losses. Based on the clinical signs, morphology, PCR amplification, and ORFV011, 059, 109, 110 and 132 gene sequences, we conclude that the isolated NA1/11 virus is a novel ORFV strain. The phylogenetic analysis revealed that NA1/11 clusters together with Xinjiang and Gansu strains. Therefore, more epidemiological surveillance is needed in China. Our continuing work to isolate and characterize ORFV isolates from different locations in China should provide valuable information about Chinese ORFV biology. In this case, comparison of the complete genome sequences between NA1/11 and parapoxvirus reference strains is necessary for better understanding of the genetic diversity and epidemiology of this complex group of viruses. Our nMDS analysis results demonstrated that the major factors causing genetic differences of ORFV strains could be geographic locations and animal hosts.

## Materials

### Sheep herds and tissue collection

The recent outbreak of orf in sheep occurred in 2011 in northeast China. It was recorded in a farm with 115 small-tailed Han sheep, including 95 breed ewes and 20 lambs aged 1 to 5 months, located in northeast Nongan county, China (124°31’ E, 44°55’ N). Skin biopsies with gross pathologic changes were collected with a surgical punch 5 mm in diameter (Miltex Stainless, Germany) and stored at −80°C for virus isolation and PCR analysis. Serum was also collected from corresponding animals and also stored at −80°C for future use. All animal procedures were reviewed and approved by the Institutional Animal Care and Use Committee at South China Agricultural University (the certification number: CNAS BL0011).

### Virus isolation

The virus was isolated from tissues collected as described with some modifications [8,30]. Briefly, 40% w/v tissue suspension in 1× MEM medium (Invitrogen) supplemented with gentamicin (50 μg/ml), penicillin (100 U/ml), streptomycin (100 μg/ml), and 5 mg/ml of fungizone were homogenized in tissue mortars. The suspensions were centrifuged at 3000 ×g for 10 min, after which the supernatants were transferred and inoculated into OFTu cells. Inoculated cells were observed daily for the presence of cytopathic effect (CPE), and passaged three times. When 60% CPE was observed, cells and media were harvested and frozen at −80°C. Polymerase chain reactions together with sequencing reactions were performed to confirm Orf virus nucleic acid specificity.

In order to isolate the virus strain, a plaque assay was carried out for further viral purification [31]. Briefly, 7×10^5^ OFTu cells per well were transferred into 6-well plates one day before infection. On the next day, the cells were infected with serial dilutions of viruses from 10^-1^ to 10^-6^. The viruses were allowed to absorb to the cells for 1 h at 37°C in a 5% CO_2_ incubator. The medium was removed and the cells were overlaid with 3 ml of MEM containing 5% FBS and 0.5% low melting point agarose (Sea Kem® GTG®, Lonza, Rockland, ME, USA). Plaques were visualized and picked at 4 or 5 dpi. A minimum of 2 or 3 plaques were picked for each isolate.

### Viral protein purification

OFTu cells were grown in T150 tissue flasks up to 90% confluency and infected with 1 MOI of NA1/11 isolate. Cells were cultured in Eagle’s minimal essential medium with Earle’s salts, supplemented with 1% penicillin-streptomycin (10,000 U of penicillin and 10 mg of streptomycin/ml in physiological saline), 1% l-glutamine (200 mM), 1% nonessential amino acids, and 10% (v/v) heat-inactivated fetal calf serum. All reagents were purchased from Life Sciences (Invitrogen). Cells were harvested when approximately 80–90% of the cells showed CPE. Debris was removed by centrifugation at a speed of 1,000 ×g for 10 min at 4°C. The supernatant containing mature viral particles was used for further sucrose gradient ultracentrifugation [32]. The purified viral particles were heat-inactivated at 96°C for 90 min and sonicated. The protein concentration was measured with Bio-Rad protein assay reagent (Bio-Rad Laboratories). The purified viral protein was frozen at −80°C for future use.

### Western blots

20 μg of total OFTu cell lysates or 2 μg of purified viral protein as described above were resolved by SDS-PAGE gel (10%) and blotted to nitrocellulose membranes. Blots were probed with orf virus infected sheep serum or Mab A3 against ORFV059 [23], followed by incubation with donkey anti-sheep or goat anti-mouse HRP-conjugated IgG antibodies (Santa Cruz) and developed using a chemiluminescent substrate ECL (Pierce-Thermo Scientific).

### Electron microscopy

The infected cell cultures of 24 hpi and the purified virions were fixed for 60 min at 4°C in a solution containing 0.1% glutaraldehyde, 4% freshly prepared formaldehyde, 1% picric acid and 3.5% sucrose in 0.1 M cacodylate buffer pH 7.2. The morphology of the virions was observed under an MFP-3D atomic force microscope from Asylum Research, Inc. [33]. For transmission electron microscopy, ultrathin sections of fixed infected cultured cells were cut on the LKB ultratome and mounted on copper grids. The sections were stained by a combination of uranyl acetate-lead citrate [34].

### DNA extraction from scab suspensions and purified virions

Tissue suspensions (200ul) or purified virion materials were prepared for viral genomic DNA isolation by using QIAamp DNA blood kit (QIAGEN, Germany) following the manufacturer’s instructions.

### Polymerase chain reaction (PCR) and sequencing

PCR was performed on DNA extracted from skin lesions, infected cell cultures, or purified virions. Five sets of primers were designed based on the OV-IA82 genomic sequence [14] to amplify the entire open reading frame of the two highly conserved genes ORFV011 (B2L) and ORFV059 (F1L) and variable genes ORFV109,ORFV110 (EEV), and ORFV132 (VEGF) localized at the right terminus of the viral genome. Interestingly, amplification of the coding region of NA1/11-ORFV132 failed using the two primers ORFV132Fw1 and ORFV132Rv1 based on the OV-IA82-ORFV132 sequence. Considering the high variation in this region, another primer (ORFV134Rv1) was designed according to the sequence of OV-IA82-ORFV134 (aa45-50). A 721 bp DNA fragment was successfully amplified, which covers the full length of ORFV132 and 150 bp of the ORFV134 coding region. The DNA sequencing results confirm that the C-terminus of NA1/11-ORFV132 is completely different from OV-IA82-ORFV132 (Data not shown). The primer sequences were as follows: ORFV011Fw1: 5’-ATGTGGCCGTTCTCCTCTATC-3’; ORFV011Rv1: 5’-TTAATTTATTGGCTTGCAG-3’; ORFV059Fw1: 5’- ATGGATCCACCCGAAATCAC-3’; ORFV059Rv1: 5’- TCACACGATGGCCGTGACCAG-3’; ORFV109Fw1: 5’- ATGGCACATAACACGTTC-3’; ORFV109Rv1: 5’- CTAACCAGACACACAAA-3’; ORFV110Fw1: 5’-ATGGGTTGCTGTAAGGTC-3’; ORFV110Rv1: 5’-TTATCCGTGCATCTGCTTC-3’; ORFV132Fw1: 5’- ATGAAGTTGCTCGTCGGC-3’; ORFV134Rv1: 5’- CACCGAGGCGGAGCCGT-3’.

PCR was carried out in a 50 μl reaction volume containing 10 μl of 5×PCR buffer (10 mM Tris–HCl and 50 mM KCl), 2 μl of DNA template, 200 μM dATP, dTTP, dCTP, dGTP, 0.4 μM of each primer, 25 μM MgCl_2_ and 0.5 μl of *Taq* polymerase (Promega Co.). PCR was performed in a thermocycler (GeneAmp PCR 2400, Perkin Elmer, Shelton, CT) for 30 cycles of denaturation at 94°C for 1 min, annealing at 58°C for 30s and extension at 72°C for 1 min 30s. PCR was ended after 10 min at 72°C. The amplified DNA products were resolved by 1% agarose gel electrophoresis and analyzed with an IS-1000 Digital Imaging System (Alpha Innotech Corp. San Leandro, CA).

The amplicons were ligated into the TA cloning Vector (Invitrogen) following the manufacturer’s instructions. Nucleotide sequencing was performed in both orientations by automated sequencing using sequencing primers T7-promotor and M13-reverse −24. Sequences were read on an automated sequencer (Applied Biosystems DNA Sequencer 373A, Norfolk, CT) and then edited using Sequencer version 3.0 (Gene Codes Corp., Ann Arbor, MI).

### Phylogenetic analysis

Using the Neighbour-Joining method, a bootstrap consensus tree was used to represent the evolutionary history of the ORFV011 [35,36]. The percentage of replicate trees in which the associated taxa clustered together in the bootstrap test is shown next to the branches [36]. Evolutionary analyses were carried out using the MEGA5 software (MEGA, version5) [37,38] and expressed based on the number of nucleotide substitutions per site. The numbers used in the phylogenetic trees represent the Geninfo Identifier (GI) sequence identification number in GenBank (http://www.ncbi.nlm.nih.gov/Genbank/). Alignment and comparison of the nucleotide and amino acid sequences among NA1/11 and reference strains were performed using ClustalW [39].

The alignment results of ORFV011 nucleotide sequences of 43 strains (all are ORFV) were applied to pairwise distance calculations using the MEGA5 software (MEGA, version5). Distance matrixes were also used for nMDS analysis below. The variance estimation method is bootstrap method (no. of bootstrap replications is 1000) and the substitution model used is Tajima-Nei model.

### NMDS

NMDS were performed based on pairwise distance matrix. The nMDS scatting plots were constructed to investigate the similarities between different ORFV strains using the free software PAST (Copyright Hammer and Harper, http://folk.uio.no/ohammer/past). The similarity measure method is Gower method. In nMDS analysis, strains were grouped by geographic isolation, animal host species, geographic isolation plus animal host species. Different groups were marked by different colors.

## Competing interests

The authors declare that they have no competing interests.

## Authors’ contributions

ZN and SL participated in design of the study. ZN, WL, SD, FG, KZ and WH isolated the virus from the clinical tissues. ZN, WL, WH prepared the data of gene sequences. ZN, SL, XL, ML and DR analyzed the data and wrote the manuscript. All authors read and approved the final manuscript.
